# Diagnostic prospects and preclinical development of optical technologies using gold nanostructure contrast agents to boost endogenous tissue contrast

**DOI:** 10.1039/d0sc01926g

**Published:** 2020-07-14

**Authors:** Priyanka Dey, Idriss Blakey, Nick Stone

**Affiliations:** School of Physics and Astronomy, University of Exeter Exeter EX4 4QL UK p.dey@exeter.ac.uk n.stone@exeter.ac.uk; Australian Institute of Bioengineering and Nanotechnology, University of Queensland St. Lucia 4072 Australia; Centre for Advanced Imaging, University of Queensland St. Lucia 4072 Australia; ARC Training Centre for Innovation in Biomedical Imaging Technology, University of Queensland St. Lucia 4072 Australia

## Abstract

Numerous developments in optical biomedical imaging research utilizing gold nanostructures as contrast agents have advanced beyond basic research towards demonstrating potential as diagnostic tools; some of which are translating into clinical applications. Recent advances in optics, lasers and detection instrumentation along with the extensive, yet developing, knowledge-base in tailoring the optical properties of gold nanostructures has significantly improved the prospect of near-infrared (NIR) optical detection technologies. Of particular interest are optical coherence tomography (OCT), photoacoustic imaging (PAI), multispectral optoacoustic tomography (MSOT), Raman spectroscopy (RS) and surface enhanced spatially offset Raman spectroscopy (SESORS), due to their respective advancements. Here we discuss recent technological developments, as well as provide a prediction of their potential to impact on clinical diagnostics. A brief summary of each techniques' capability to distinguish abnormal (disease sites) from normal tissues, using endogenous signals alone is presented. We then elaborate on the use of exogenous gold nanostructures as contrast agents providing enhanced performance in the above-mentioned techniques. Finally, we consider the potential of these approaches to further catalyse advances in pre-clinical and clinical optical diagnostic technologies.

## Abstract 2

Optical biomedical imaging research utilising gold nanostructures as contrast agents has advanced beyond basic science, demonstrating potential in various optical diagnostic tools; some of which are currently translating into clinical applications.

## Graphical abstract

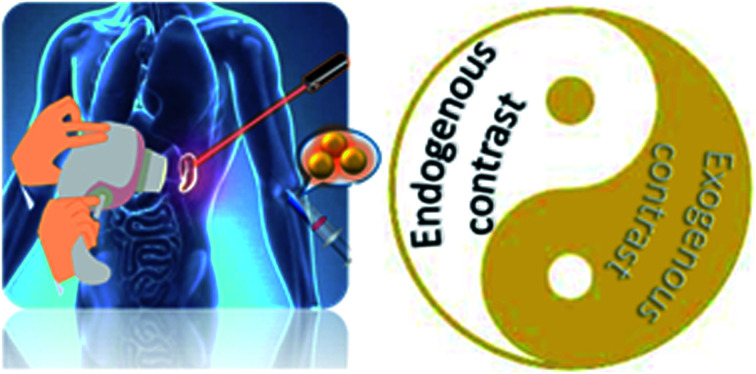

## 1. Introduction

The term “cancer theranostics”^1–3^ is often broadly associated with a combination of cancer targeting strategies, contrast agents and therapeutic agents. The term was originally coined suggesting a cocktail of components packaged into a single platform that could target a specific cancer, accumulate in the tumour, with the detection modality helping to “see” the tumour, thereby diagnosing the cancer-type and tumour location. This would then be followed by either spontaneous, time-dependent or externally triggered release of the drug payload or generation of heat to perform localized therapy.^4^ Even with exponential growth in numbers of research articles, the delay in translational phase of various cancer theranostics can be debated to be due to: the lack of information provided in literature reports,^5^ poor reproducibility of structure and performance,^6,7^ insufficient standardization of methodologies for performance comparison across and within various modalities,^6,7^ and legitimate concerns regarding whether the optimal nanostructure for therapy was also optimal as a contrast agent.^8^ Such concerns invariably imply that while theranostics are the ultimate goal and use of nanostructures that can act as contrast agents and aid in therapy is important, structure dependent performance optimization might need to be carried out independently for detection and therapy. Gold nanostructures have much to offer, with optical properties that can be tailored to allow use in multiple optical imaging modalities as exogenous contrast agents, as well as benefiting from photothermal conversion which can facilitate photothermal therapy.^9^ In this review, we focus on the promising advances in both gold nanostructure contrast agents and optical modalities while critically discussing the clinical prospects of their allied strengths. The next few paragraphs provide a brief overview of the concepts and correlation of gold nanostructures, optical detection techniques and the use of near infrared (NIR) light for detection through tissue.

**Fig. 1:**
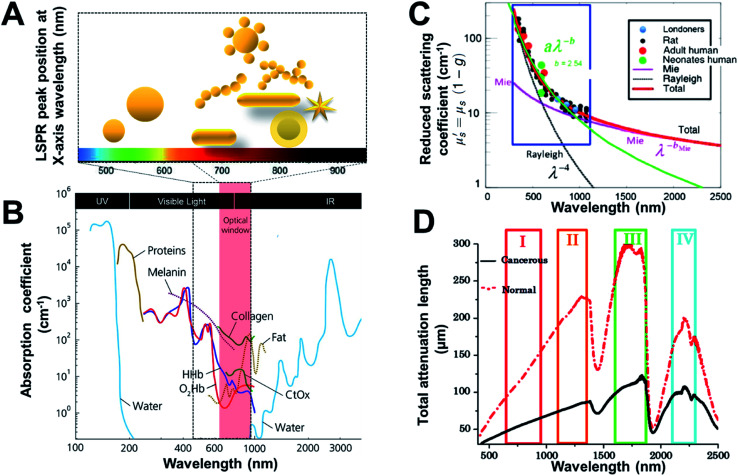
Wavelength-dependent optical properties of tissue and gold nanostructures. (A) Light penetration through tissue and absorbance LSPR of gold nanostructures (depicted in cartoon representation). (B) Optical window with absorbance of biomolecules in relative proportions they are present in body. The spectra are given with respect to the specific concentration in mM,^27^ (C) Wavelength dependence of reduced scattering coefficient, for human and rat skin; dots present experimental data, and solid and dotted lines represent theoretical dependences from Mie and Rayleigh theories,^21^ and (D) spectra of the total attenuation lengths (μm) from normal and cancerous prostate tissues using the I, II, III, and IV NIR optical windows.^20^ Reproduced from ref. 20 and 27 with permission from [SPIE], copyright [2014 and 2019]. Reproduced from ref. 21 with permission from [Elsevier], copyright [2014].

Gold nanostructures have fascinating optical properties that originate from localized surface plasmon resonances (LSPR) that are generated when they interact with light. The wavelength of the LSPR peak for gold nanostructures can be typically tuned within the visible region to the near infrared (NIR) and can be specifically controlled by varying factors such as size, shape and degree of aggregation.^10–13^ In addition, they can also offer very high Rayleigh scattering cross-sections and are not subject to photobleaching. Gold nanostructures demonstrate structure-dependent tunability of the LSPR in visible to NIR regions as shown in Fig. 1A. They can be easily functionalized with a variety of functional molecules or macromolecules that possess a thiol or other gold anchoring functional group including polymers, biomolecules or organic molecules.^14,15^ This enables stability from aggregation, biocompatibility, molecular encoding and molecular targeting. All these factors make gold nanostructures amenable to be used as exogenous contrast agents in optical modalities. We will explore critical features relevant to each optical technology in their respective sections.

Photons from the excitation light source interact with tissues and its various components resulting in high absorption and scattering leading to loss of photons reaching the light-sensitive detectable molecule/component embedded in the tissue (minimal penetration). Noticeably, NIR light benefits from higher penetration into tissues due to minimal absorbance (low absorption coefficient) by tissue components is often referred to as the “tissue transparency window” or “optically clear window” (as shown in Fig. 1B).^4,16,17^ NIR also has a relatively low risk of damaging living tissues compared to visible or UV wavelengths, due to the lower photon energy of NIR and the significantly reduced absorbance by tissue components, resulting in NIR technologies dominating the field. The NIR region can be divided into the NIR I (650 nm–950 nm) and NIR II (1000–1350 nm) regions where the former is more commonly used due to the availability of optics and low noise filters and detectors.^18,19^ While higher tissue penetration depth may be achieved by NIR II as the scattering is reduced (Fig. 1C), thereby improving the total attenuation length (Fig. 1D).^20,21^ NIR-active gold nanostructures^22,23^ can efficiently absorb or scatter NIR light and therefore provide optical contrast within tissues and cells. However, the geometry and size of the gold nanostructures impact on the wavelength of the plasmon coupling (LSPR). Therefore, careful design considerations are required for gold nanostructures to make them optically active in the NIR (as shown in Fig. 1A). This is attained by providing increased absorption and maximum plasmon resonance activity in the NIR, making them highly effective NIR contrast agents.

NIR optical technologies under development for *in vivo* diagnostic imaging should enable either fast, high spatial resolution visualization of tissues or highly specific detection of the molecular changes associated with disease. Although NIR light penetrates deeply into tissues, it is highly scattered, with around one scattering event every 0.1 mm.^17^ This reduces the prospects of imaging at depths beyond a few mm with conventional optics and detectors, due to the scrambling of the location of the signal. However, it still allows for detection of signals of interest from depths of many cm. A myriad of NIR detection methodologies, considering both endogenous and exogenous contrast, are at varying stages of clinical translation from early feasibility, to *in vivo* animal testing, to use in humans.^4^ For example, optical coherence tomography (OCT) is an established clinical modality that gives cross-sectional images of tissues and is widely used to image the retina in ophthalmology. OCT has cellular-scale resolution, but due to significant scattering effects the imaging depth is limited to approximately 2 mm. On the other hand, Photoacoustic imaging (PAI)^24^ and Spatially Offset Raman spectroscopy (SORS) imaging^25,26^ can both be categorized in the translational phase from research development towards pre-clinical and clinical studies. They have been tested on humans and demonstrate great promise for rapid clinical imaging or detection of abnormal diseased tissues. These techniques overcome tissue scattering, in fact SORS benefits from it, enabling deeper detection where there is a trade-off between penetration, depth detection and spatial resolution depending on the particular optical setup. These techniques can be utilized to visualize endogenous tissue or tumour features (inherent signal from various components/parts of the tissue), however the scope and utility can benefit significantly from exogenous contrast agents (external agents that need to be injected into the body/tissue), such as gold nanostructures. Hence, reflecting on the technological advancements of the promising optical diagnostic modalities coupled with the employed gold nanostructure contrast agents is critical.

## 2. Optical coherence tomography (OCT)

OCT measures the interference between two low-coherence beams of light, a reference beam and sample beam, after it is backscattered by the tissue. The technique is capable of 2–10 μm resolution at depths up to about 2 mm. The two main instrumental configurations either use a broadband non-coherent source or a swept coherent laser source. The endogenous contrast is primarily generated by the different scattering properties of tissue, which has made it particularly useful in identifying different layers of cells in the retina and coronary arteries where high levels of contrast are observed. However, other tissue types typically lack good endogenous OCT contrast, so there has been a need to develop exogenous agents to expand the utility of OCT.

The use of gold nanostructures as contrast agents leads to an increased signal to noise ratio and hence depth of penetration (with sufficient signal recovery). For example, Huang *et al.* used gold nanorods to enhance the OCT of mammalian embryos, where the improved contrast enhanced visualization of the organ periphery and also increased the imaging depth.^28^ Studies optimizing the size or shape of the gold nanostructures have shown that structures featuring the highest scattering at the OCT source wavelength provided the best contrast.^29,30^ For example, for an OCT light source with a centre wavelength at 930 nm, nanostars with an LSPR peak maxima at 830 nm (prepared with 120 nm seed gold nanoparticles NPs) which exhibited higher scattering cross-sections at 930 nm provided better contrast as compared to nanostars which had LSPR maxima at lower wavelengths (prepared with smaller diameter seed NPs).^30^ Additionally, it was found that nanoshells gave significantly improved scattering and OCT signal compared to gold nanorods, when using an OCT source with a wavelength centre of 1320 nm, owing to their LSPR peak position.^29^

Much of this work has been of a fundamental nature to demonstrate the utility of gold nanostructures to enhance imaging contrast. Some reports have shown where gold nanorods^31^ and gold nanoprisms^32^ have been used to enhance the amount of vasculature that could be imaged in mice. In particular, Si and coworkers^32^ conducted imaging studies of the vasculature in a melanoma tumour model, where it was possible to image the tumour microvasculature which allowed better characterization of regions of ischemia. This enhancement in performance makes this a promising approach for assessing the response to antiangiogenic therapies^33^ where imaging of the microvasculature is crucial, however this is yet to be demonstrated in practice.

### 2.1. Multiplexed OCT

**Fig. 2:**
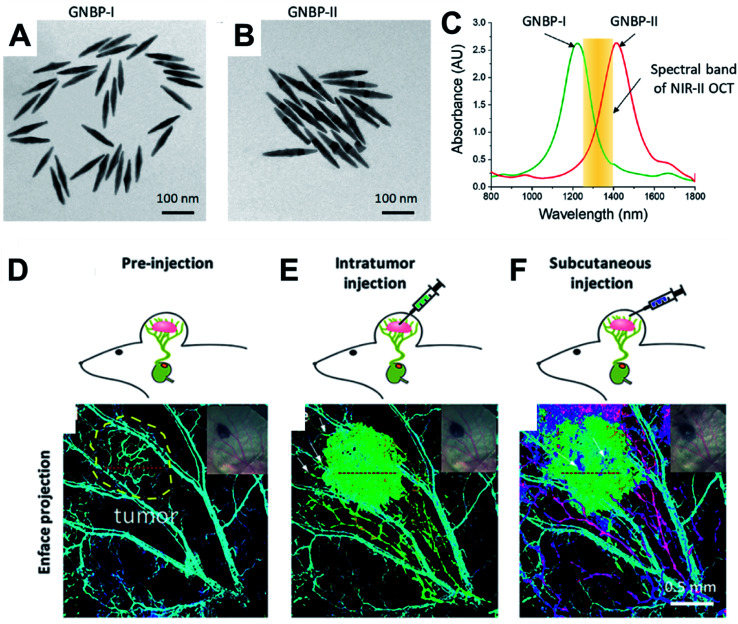
TEM micrographs of gold nanopyramids with average lengths of (A) 133 nm GNBP-I and (B) 177 nm GNBP-II. (C) Optical spectra showing the LSPRs of GNBP-I and GNBP-II, which lie on either side of the OCT spectral band in the NIR II region. (D) OCT showing endogenous contrast of vasculature in the region of a tumour, (E) OCT showing additional exogenous contrast after injecting GNBP I intratumourally, (F) OCT showing exogenous contrast generated by injecting GNBP-II subcutaneously allowing visualization of the lymphatic system. Reproduced from ref. 34 with permission from [The American Chemical Society], copyright [2019].

Si *et al.*^34^ also reported multiplexed contrast agents that were able to trace separate lymphatic flows from a melanoma tumour simultaneously. They synthesized two sets of gold nanopyramids with average lengths of 137 and 177 nm (Fig. 2A and B) which resulted in narrow LSPRs with maxima at 1225 nm and 1415 nm respectively (Fig. 2C), which were situated either side of the centre wavelength of the OCT source (1320 nm). In Fig. 2D, the pre-injection control image the endogenous OCT contrast that allows the vasculature in the region of a tumour to be visualized. Intratumoural injection of GNBP-I allowed imaging of the tumour due to diffusion of the nanostructure as well as lymphatic drainage. Subcutaneous injection of GNBP-II was then used to allow the lymphatic vessels to be further characterized where peritumoural and intra tumoural lymphatic vessels could be visualized. The multiplexed imaging was enabled using a custom spectral analysis algorithm, which allowed deconvolution of the spectral signal to provide exogenous spectral contrast that enabled visualization of the separated lymphatic flows (Fig. 2E and F). The ability to dynamically image different aspects of lymphatic system is particularly important in cases of lymph node metastasis. For example, by labelling the nanostructures with different targeting biomolecules multiplexed OCT opens the opportunity for it to be used as a preclinical tool that could be used to assess heterogeneous tumour cells that express different receptors or to assess the different endogenous receptors present in the lymphatic system.

### 2.2. pH dependent OCT

**Fig. 3:**
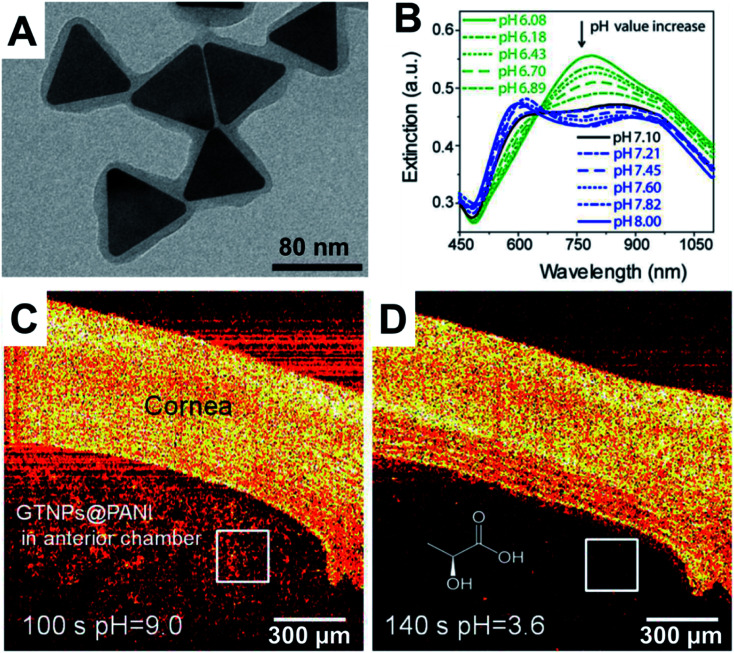
(A) TEM micrograph of polyaniline (PANI) coated gold triangular nanoprisms. (B) Change in optical response as a function of pH. (C) OCT imaging of cornea and anterior chamber of a crucian carp at pH 9 and then (D) at pH 3.6. Reproduced from ref. 37 with permission from [The American Chemical Society], copyright [2017].

Xiao and coworkers^35,36^ prepared gold NPs with “smart” small molecule ligands that allowed the OCT signal to switch on when the pH was decreased from 7.4 to 5.5. At a pH of 7.4, the ligands gave rise to a negative charge due to a terminal carboxylic acid, which provides colloidal stability *via* electrostatic repulsion. These nanostructures have an LSPR at around 520 nm and do not have a strong OCT signal due to a lack of an LSPR in the NIR region. At pH 5.5, a proportion of the ligands undergo hydrolytic cleavage of an amide bond, resulting in loss of the carboxylic acid and formation of a primary amine. This yielded NPs with a mixed monolayer of ligands with a proportion of intact ligands with a terminal carboxylic acid and a proportion of cleaved ligands with a terminal primary. At pH 5, the carboxylic acids are negatively charged and the amines are positively charged, such that electrostatic attraction results in aggregation of the gold NPs and the appearance of a broad LSPR in the NIR region due to plasmon coupling. This pH-switchable LSPR in the NIR resulted in a strong pH-dependent OCT signal. This switching of the OCT signal has been demonstrated *in vitro* using NPs incubated with HeLa cancer cell line. More recently Tang and coworkers^37^ prepared gold triangular nanoprisms coated with polyaniline (PANI) that were able to assess the pH in the anterior of the fish eye. At pH 1–6, these NPs had an absorbance maximum at 760 nm, while at pH 8–14 the spectrum changed to have maxima at 600 nm and 900 nm with significantly reduced absorbance at 760 nm. This switch in the spectra occurred in the pH 6–8 range and was found to be reversible and detectable by OCT. This could therefore be employed with OCT imaging to assess pH changes in a phantom that contained a tumour mimic, as well as *ex vivo* in the eye of a crucian carp. Such pH sensitive OCT contrast agents are relatively early in the development pipeline, where the next steps for *in vivo* imaging in animal models would benefit inclusion of targeting agents to mediate the biodistribution and pharmacokinetics (Fig. 3).

## 3. Photoacoustic imaging (PAI) and multispectral optoacoustic tomography (MSOT)

PAI combines the modalities of optical and ultrasound imaging by using pulsed lasers to illuminate tissue, causing transient heating of absorbing species. The temperature rise results in thermoelastic expansion, producing acoustic waves that are detected using ultrasonic transducers. The ultrasound signal is then mathematically reconstructed to produce the images. Due to sound waves having lower absorbance and scattering in tissues, the detection of ultrasound rather than photons helps to mitigate issues with photon scattering, where imaging depths of greater than 4 cm are possible with a 255 μm resolution.^38^ It should be noted however, that the precise resolution and depth of imaging is ultimately dependent on the illumination optics and the detector configuration, where there is typically a trade-off between spatial resolution and depth of penetration. Early PAI instruments used single wavelength NIR lasers for illumination. The optical absorbance of endogenous biomolecules varies significantly in the red and near infrared regions, such that the different distribution of these biomolecules provides endogenous contrast for PAI. A recent advance is the use of rapidly tuneable lasers which allow illumination with multiple wavelengths on a millisecond timescale and is often referred to as multispectral optoacoustic tomography (MSOT). Using spectral unmixing algorithms it is possible to differentiate endogenous biomolecules with different NIR absorption maxima. Instruments with a laser that is tuneable between 660–980 nm are able to differentiate and produce separate images of oxygenated haemoglobin and deoxygenated haemoglobin. This capability when expanding the window up to 1300 nm also allows lipid and melanin to be distinguished.^39^ Ultimately MSOT provides fast, high resolution *in vivo* molecular imaging from deep inside biological tissue and our review will primarily focus on gold nanostructure-based contrast agents for this technique.

### 3.1. Exogenous contrast agents in MSOT

Addition of exogenous species such as NIR absorbing gold nanostructures can further enhance the image contrast. In the case of gold nanostructures, high NIR absorbance results in higher thermal expansion and improved acoustic output. Furthermore, if these can be functionalized to specifically bind to cells of clinical interest, then the contrast from an altogether different tissue and or pathology can be achieved. Nanorods and nanostars have often been used due to the ability to tune LSPR peak position into the NIR by adjusting their structural parameters. Typically, the nanostructures are passivated with a silica or polymer coating to prevent aggregation, because this would result in plasmon coupling and a shift of the LSPR away from the desired wavelength. For example, Taruttis and coworkers were able to image cardiovascular dynamics using circulating gold nanorods stabilized with a proprietary polymer.^40^ Comenge and coworkers^41^ demonstrated that a 35 nm silica shell was enough to protect gold nanorods from aggregation and restrict plasmon coupling when they were used to label stem cells. They were then able to track these cells that were labelled with the nanorods after subcutaneous injection into mice and did not observe significant spectral broadening for up to 15 days.

In a follow up study, Comenge and coworkers^42^ found that cell division and apoptosis (cell death) caused a loss of the gold nanorod signal over extended periods of time, which is an issue for longitudinal cell tracking studies that use nanoparticles or synthetic dyes. To overcome this, they have genetically engineered the stem cells to express a NIR fluorescent protein that can act as a second exogenous MSOT reporter. When these genetically modified cells were labelled with the gold nanorods, the gold nanorods allowed the initial biodistribution of the cells to be tracked, where at the early stages the concentration of the fluorescent protein was too low to be observed by MSOT. However, as the stem cells formed tumours the levels of the fluorescent protein increased and allowed the tumours to be imaged up to 40 days. This was also complimented by using whole body bioluminescence imaging in conjunction with the NIR fluorescence to assess tumoural distribution in the mice.

The ability for MSOT to rapidly acquire images across the NIR spectrum can also be exploited to enable multiplexing of signals obtained from contrast agents that have varying LSPR maxima. Bayer and coworkers^43^ prepared silica coated nanorods with differing aspect ratios to give LSPR maxima at 780 and 830 nm. The 780 nm LSPR nanorods were then bio-conjugated to monoclonal HER2 antibodies, while the 830 nm LSPR nanorods were bio-conjugated to monoclonal EGFR antibodies. Cell lines with HER2 and EGFR receptors when exposed to the respectively-targeted nanostructures were taken up, following which they were prepared into a tissue phantom and were successfully imaged providing multiplexed signal. Wang and co-workers have used the differing position of the plasmon resonances of nanostars and nanorods stabilized with chitosan to follow the differing biodistribution and pharmacokinetics of the two contrast agents after injection into a mouse.^44^

### 3.2. Multimodal imaging that incorporates MSOT

**Fig. 4:**
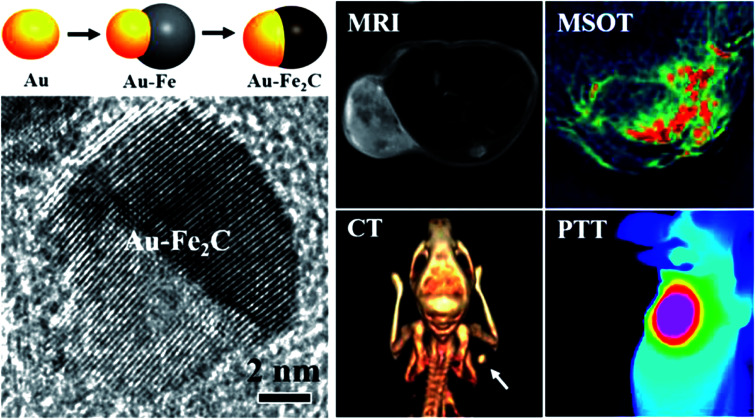
Schematic of the preparation of Janus Au–Fe_2_C nanoparticles and high resolution TEM micrograph. Comparison of the MRI, MSOT, CT and PTT imaging using the Janus Au–Fe_2_C nanoparticles as contrast agents. Reproduced from ref. 45 with permission from [The American Chemical Society], copyright [2017].

The versatility of gold nanostructures to be functionalized and self-assembled has created opportunities to prepare multimodal contrast agents, which utilizes the advantages of different detection modalities. Ju and coworkers^45^ have prepared Au–Fe_2_C Janus nanoparticles (see Fig. 4) which featured a broad LSPR in the NIR region, and exhibited transverse relaxivities (*r*_2_) that were higher than Resovist a commercially available MRI contrast agent, acting as an efficient negative MRI contrast agent. The gold component provided the CT imaging capability as well as photothermal treatment (PTT) potential. The Janus nanoparticles were then coupled to affibody proteins with a strong affinity to the HER2 receptor and injected into mice bearing a HER2 expressing tumour model. They were successfully able to localize the contrast agents accumulating in the tumours using MRI, MSOT and CT as shown in Fig. 4. The multimodal nature of these agents provides flexibility in choice of biomedical imaging technology for preclinical studies where the resolution of the imaging progressively increases going from CT, to MRI and to MSOT.

Wang and coworkers^46^ prepared a MnO_2_–Au hybrid material that was stabilized with hyaluronic acid. The hybrid material had a broad LSPR signal that extended into the NIR to give contrast in MSOT and the magnetic properties of MnO_2_ affected the T1 relaxivities (*r*_1_) of water making it a positive contrast agent. The high electron densities of the metals also allowed for CT contrast. Multimodal imaging was demonstrated in a mouse xenograft tumour model, where the CT and MRI modalities allowed the tumour to be localized. Furthermore, it allowed the research team to gain a broad assessment of the biodistribution and then MSOT was used to obtain high resolution imaging of the tumour over time. In addition, the MnO_2_ had a therapeutic effect when in contact with hydrogen peroxide which is up-regulated in tumours to generate oxygen to alleviate hypoxia and regulate pH. By taking advantage of the endogenous contrast between oxygenated and deoxygenated haemoglobin it was possible for MSOT to monitor this therapeutic effect. The synergy of this effect with photothermal therapy afforded by the gold was also investigated.

Neuschmelting and coworkers^47^ prepared silica coated nanostars labelled with IR780 to prepare MSOT-SERRS contrast agents (dedicated section on SERS to follow). These agents were injected into a glioblastoma tumour model that was clearly visible by endogenous tissue contrast using MRI but not *via* MSOT prior to injection. Within 1 minute of injection, the MSOT signal showed clear accumulation in the tumour as well as being able to image the areas that contained oxygenated and deoxygenated haemoglobin. This demonstrates the potential for rapid and information-rich imaging in glioblastoma surgery with clear delineation of the tumour periphery. The pharmacokinetic profiles were further monitored over 800 minutes in the tumour, intravascularly and in healthy tissue. After euthanizing the animals, high resolution ex vivo mapping of tumour sections were performed by SER(R)S (*i.e.*, resonance SERS) at different time points to demonstrate the distribution of the contrast agent within the tumour as a function of time after injection. While these SERRS mapping studies were performed ex vivo on tissue sections they corroborated the observed MSOT *in vivo* imaging. In a clinical setting, the ability of imaging during glioblastoma surgery to guide tissue resection, followed by analysis of the resected tumour *via* SERRS, could help ensure that incursion into healthy brain tissue is minimized.

## 4. Raman spectroscopy (RS)

Raman spectroscopy has been traditionally used for characterizing known or unknown molecules by decoding chemical structure and lately as a label-free modality for detection of disease markers or chemical contaminants.^48,49^ It has been extensively used in biomedical optical detection due to the inherent differences in fluids, cells and tissue constituents like proteins, lipids, nucleic acids, carbohydrates, water and others; which has been found to vary in their proportions in healthy and cancer tissues.^50–52^ Raman techniques have been employed in clinical research to date in biopsy tissue sections and at the surface of the organs in multiple cancer types like epithelial, breast, head and neck, lung and oesophageal cancers which inevitably demonstrates its competence as a diagnostic modality.^53–55^ Furthermore, Raman spectroscopy has been demonstrated, in concert with multivariate statistical analysis or machine learning, to be effective in identifying primary and secondary cancers in the lymph nodes,^54^ in the head and neck and in axillary breast nodes at point of care in the operating suite.^56^ More recently, calcium hydroxyapatite has been extensively investigated in breast cancer tissues as a constituent of type II micro-calcifications both in large surgically resected *ex vivo* breast tissue (employing specialized Raman instrumentation, discussed in the following sub-sections) and biopsies.^57^

Use of standard spontaneous Raman mapping can provide significant detail of location and intracellular compositional changes. However, time to acquire images for even small areas of tissue (2 × 2 mm^2^) can take many hours. Compressive sensing of spontaneous Raman scattering and coherent Raman imaging has gained momentum in attempting to accelerate image acquisition to speeds sufficient for pathologists to utilize the endogenous contrast provided by these approaches to provide detailed structural imaging of cells or tissue sections often up to few tens to hundreds of micrometres in thickness.^57,58^*In vivo* Raman diagnostics providing endogenous tissue contrast have been demonstrated in clinical trials, but usually require access either to the surface of the skin, or the lining of hollow organs *via* optical fibers. Examples are endoscopic probes for Raman laser illumination and collection from inside the body for example in oesophagus cancers^53^ and brain cancers.^59^ More recently needle probes have been developed for probing disease specific molecular changes in solid organs.^60^ Of course the ideal method for sampling the Raman signals would be non-invasive. The utilization of the properties of NIR photon migration in tissues allows the principle of spatially offset Raman spectroscopy to probe native biochemical signals from depths in tissues of up to 4 cm.^61^ However, signals measured in this way are weak and ideally need a strong disease specific contrast such as that demonstrated from hydroxyapatite. An alternative to this is to provide a bright Raman signal with a spectrum highly distinguishable from the surrounding tissues. This would not only need an improvised Raman instrumentation strategy but would need to exploit the advantages of gold nanostructure-based contrast agents. With suitable targeting ligands, exogenous contrast agent could reach a specific cancer type and provide diagnosis. This is possible when employing relevant pairs of targeting ligand-Raman tag on gold nanostructure contrast agents.

### 4.1. Surface enhanced Raman scattering (SERS) gold nanostructures

**Fig. 5:**
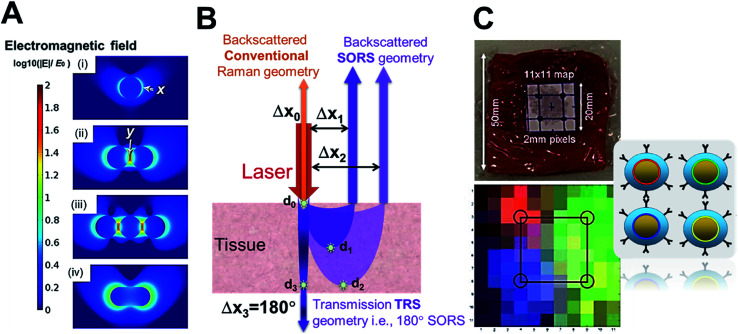
(A) Comparison of hot-spots and electromagnetic field around NPs and at nano-junctions,^75^, (B) demonstration of SORS set-up with Δ*x* spatial offset correlated to *d* detection depth which approximately provides the highest signal information, and (C) demonstration of SESORS (TRS) detection with Oxonica silica-coated SERS-tagged 100 nm NPs through 5 cm of *ex vivo* pork tissue with multiplexed detection (4 tags, depicted as 4 colours). Reproduced from ref. 75 with permission from the [Beilstein Institute for the Advancement of Chemical Sciences (Germany)], copyright [2016]. Reproduced from ref. 25 with permission from [The Royal Society of Chemistry], copyright [2011].

In contrast to the above, tagged Raman spectroscopy finds use in *in vivo* disease detection using tagged gold nanostructures as contrast agents.^48,62^ The gold nanostructures act as Raman signal amplifiers due to the SERS phenomenon which has been reported to provide a signal enhancement of 4–10 orders of magnitude.^63,64^ This phenomenon occurs when molecules with high Raman cross-sections (often small aromatic molecules referred to as Raman or SERS tags) are positioned at the surface of plasmonic metals like gold nanoparticles (NPs) and/or between nano-junctions where the electromagnetic field is strong. The NP morphologies (shapes, sizes and assembly structures) also dictate the plasmon coupling which influences the position of the LSPR, which upon approximately coinciding with the laser line can boost the SERS enhancement.^63^ The electromagnetic field intensity around a single plasmonic gold NP is often classified as first generation hot-spots (Fig. 5A ‘*x*’), whereas, NP–NP junctions in nano-assemblies are referred to as second generation hot-spots (Fig. 5A ‘*y*’) where, the intensity of *y* is often greater than that of *x*, suggesting nano-assemblies can play a crucial role in SERS signal amplification. Thus higher SERS signal enhancement would be obtained when a tag molecule sits at *y* rather than *x*. Pioneering reports from the groups of van Duyne, Liz-Marzán and Baumberg have dealt with critical concepts of electric field distribution, hot-spot density, SERS enhancements and SERS tag orientation in the hot-spot.^64–70^ In addition, studies from Dey and co-workers delineating crucial SERS tagging factors have come to light that reports the importance of the sequence of tag incorporation^71^ as well as hot-spot and tag positioning^72^ on SERS signal maximization. Additionally, the importance of choosing the Raman or SERS tag is also crucial and readers are directed to some of the recent reviews addressing that.^62,73,74^ In summary, SERS enhancement factors (EF *i.e.*, enhancement of the tag signal intensity at a certain concentration when on a nanosurface compared to the pure form) are dependent on multiple factors including nanostructure design, gold nanostructure and SERS tag concentration, laser line used in relation to the LSPR, laser power at the sample and Raman volume probed depending on the instrumental set-up used.

### 4.2. Spatially offset Raman spectroscopy (SORS)

Standard confocal Raman instrumentation, has been used to demonstrate the benefit of gold nanostructures as Raman SERS contrast agents. Qian *et al.*^76^ have reported this *in vivo* with SERS NPs using Rhodamine 6G as the Raman tag. They demonstrate the higher spectral sensitivity of optical Raman spectroscopy as compared to optical fluorescence spectroscopy. Ou *et al.*^77^ have studied duplexed SERS detection using gold nanostars both *ex vivo* and *in vivo*. The tissue scattering of both the incident laser and emitted Raman photons has been a challenge in promoting Raman SERS spectroscopy as a disease/tumour detection technology, utilizing standard epi-collection approaches, limits tissue sampling to around 200 μm in depth. The development of modified Raman instrumentation, known as backscattered spatial offset Raman spectroscopy (SORS) first reported by Matousek *et al.*,^78^ has been game-changing in the field allowing detection of sub-surface generated Raman photons (signals from within depth) by spatially offsetting the excitation and collection point (as opposed to confocal systems where these two coincide) as shown in Fig. 5B. It has been shown that increasing the offset employed results in signal recovery from deeper within the tissue as shown in Fig. 5B where the offset Δ*x*_*n*_ correlates to the *d*_*n*_ depth (marked with a star) at which highest signal information is obtained for that set-up.

### 4.3. Surface enhanced spatially offset Raman spectroscopy (SESORS)

**Fig. 6:**
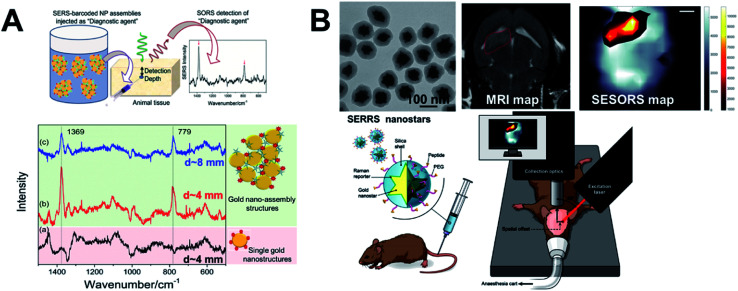
SESORS diagnostics and gold nanostructures as contrast agents. (A) Demonstration of benefit of using tailored sub-100 nm nano-assemblies compared to single NPs with SESORS detection through 8 mm of *ex vivo* chicken tissue,^81^ and (B) demonstration of silica-coated sub-100 nm nanostars for *in vivo* SESORS detection through skull.^15^ Reproduced from ref. 81 with permission from [Wiley Materials], copyright [2013]. Reproduced from ref. 15 with permission from [Ivyspring International Publisher], copyright [2019].

SESORS *i.e.*, signals from SERS nanostructure (gold nanostructure functionalized with the tag) contrast agents, detected with SORS instrumentation, allows us to overcome the limited contrast in soft tissues and with stronger signals allows greater depths to be probed.^25^ Much of the focus in the development of SESORS has been in demonstrating the capabilities of the SORS technique. This has also led to the use of *ex vivo* tissues of different animal models, as mice models are limited in their ability to provide human relevant depths for photons to travel through. Stone *et al.*^25^ demonstrated the first SESORS measurement by utilizing transmission Raman spectroscopy (TRS) detection, as a special case of SORS, where the sample was excited at one surface and the Raman photons collected in transmission through the tissue from the other surface (as shown in Fig. 5B similar to a scenario with 180° SORS offset). They used commercial NPs from Oxonica which were gold NP cores with a 100 nm diameter, tagged with SERS tags and then encapsulated with silica shell for stability, featuring an LSPR in the visible. Employing the SERS tagged NPs (∼1.8 × 10^9^ NPs) in a TRS set-up with an 830 nm laser line providing 290 mW power at the sample surface, the SERS signal could be detected through 5 cm of pork tissue. In addition, they have also demonstrated identification of four different tags with high accuracy, thereby reporting multiplexed detection with SE-TRS from highest depths achieved to date, as shown in Fig. 5C. This was followed by important contributions from van Duyne's group employing SESORS detection using a system with 2–3 mm offset, 785 nm excitation with 300 mW power to detect NPs (9 × 10^13^ NPs) with *trans*-1,2-bis(4-pyridyl)-ethylene (BPE) SERS tag through *ex vivo* bone of 8 mm thickness^79^ and detecting glucose *in vivo* in a rat model using silver nanostructures.^80^ Furthermore, Dey *et al.*^81^ employed custom-made gold nano-assemblies with tailored LSPR into the NIR and further tagged them with 2-quinolinethiol as the SERS tag for probing detection with backscattered SESORS set-up. Employing a fixed offset (Δ*x*) of 2 mm and 785 nm laser line (delivering 400 mW power), they demonstrated detection at 8 mm of *ex vivo* protein-rich chicken tissue and 1–2 mm of lipid-rich chicken tissue with high signal to noise ratio. As shown in Fig. 6A, they have shown improved performance for gold nano-assemblies when compared to single gold NPs at identical gold concentrations and injection depths, thus suggesting the benefit of gold nano-assemblies as contrast agents. Nicolson *et al.*^15^ studied *in vivo* SESORS detection using modified single nanostructures specifically, nanostars. The nanostars exhibited a LSPR peak maxima at 621 nm, which was then functionalized with a NIR-absorbing SERS tag IR792 to provide a further amplified resonance SERRS effect. Such targeted nanostars (100 μL of 8 nM) were then detected *in vivo* in a mouse brain cancer model through the skull using 785 nm laser (∼130 mW power), as shown in Fig. 6B, where the tumour margin observed by the SESORS measurements was additionally validated by comparing with MRI maps. Such reports demonstrate the clinical prospects for SESORS detection, although extensive toxicity evaluations for each of the nanostructures will be necessary before they can be safely used in humans.

Instrumentation development and miniaturization has a tremendous effect on the technology advancement and market penetration. A handheld SORS instrument,^26^ an endoscopic Raman fiber bundle^82^ are examples enabling pre-clinical and clinical studies respectively. Similarly advantageous is the use of flexible inverse-SESORS with a ring beam illumination and point collection enabling the use of higher laser power illumination (for the same power density as a central spot) for improved sensitivity and increased detection depths. The group of Matousek and Stone have also deployed the use of training sets, models and algorithms to be able to detect the depth more accurately by measuring SERS signals from external surfaces and eliminating the need of *a priori* data at multiple depths.^83,84^ This provides evidence that these approaches could provide depth measurements that would enable their use as minimally invasive clinical diagnostic tools.

The functionality of the SERS tags themselves to provide a measure of the physical or chemical properties of their surroundings has also been researched. Utilizing a stimuli-responsive SERS tag like methyl benzoic acid (MBA) onto a 100 nm gold NP, Gardner *et al.* have reported the minimally invasive detection of pH in the range of 2–10 by monitoring structural change (Raman detectable) of the tag with pH (pH-SESORS)^85^ and temperature change of up to 20 °C by monitoring the Anti-Stokes to Stokes Raman signal ratio of the tag as a function of temperature (T-SESORS).^86^ Cancer tumours are known to have acidic pH environments and when treated with photothermal therapy the temperature increase at the tumour local environment is critical for the therapy to be effective, making the above reports stepping stones in the direction of clinical translation. Therefore, such technological advancements promise a bright future for Raman biomedical diagnostics with gold nanostructures as a contrast agent.

## 5. Key design considerations of gold nanostructure contrast agents

With extensive understanding of the NIR detection technologies and their translational prospects, it is prescient to dig deeper into the design of gold nanostructure contrast agents. Ideally, an *in vivo* diagnostic agent (*i.e.*, contrast agent with biocompatibility and targeting functionality) would need to have a high blood circulation lifetime, feature minimal toxicity to normal cells, provide high specificity (specific targeting and accumulation) for abnormal *versus* normal tissues and after performing its function be cleared out from the system *via* a time-dependent or externally triggered mechanism. These features cumulatively affect the diagnosis performance. In particular, the key features of a contrast agent are to provide high signal to background (imaging contrast), be minimally toxic and provide possibilities of multiplexing. Of major importance in design and clinical translation of contrast agents are process, product and functional reproducibility and process scalability. Furthermore, major discrepancies in standardization of optical response or performances, including but not limited to, optical density or absorbance, SERS signal enhancement factors, have severely delayed the selection of deserved candidates for pre-clinical and clinical trials. This complex balancing of factors has thus been a major bottleneck, resulting in gold nanostructure contrast agents being stuck in the research phase for an extensive period of time. Simpler gold nanostructures require fewer synthesis steps, thus making it easier to scale up the process while maintaining batch-to-batch reproducibility. Nevertheless, simple scalable and reproducible gold nanostructures like spherical gold nanoparticles may not provide optimal functionality. This leads us to discuss the design considerations that influence the optical responses critical for enhanced contrast and detection.

The performance of gold nanostructure contrast agents for OCT, PAI, MSOT and SESORS primarily relates to absorbance cross-section, NIR LSPR absorbance peak, plasmon coupling, electromagnetic field intensity (hot-spots), hot-spot position and hot-spot density. Specifically, and importantly, the nanostructure LSPR profile and its plasmon response at the NIR laser excitation wavelength is the key optical property that translates into respective characteristic detection responses. This has thus made nanorods, nanoshells and nanostars attractive for use. Nanorods typically feature a transverse LSPR around 520 nm and a significantly more intense and narrow longitudinal LSPR in the range of 600–1000 nm,^87^ while absorbing 3–5 times more light energy at the longitudinal LSPR than the spherical gold nanoparticles (NPs) (visible LSPR 510–540 nm) of similar gold mass. In contrast, nanoshells feature a broader NIR LSPR centred around 700–800 nm.^88,89^ The larger size (often 50–200 nm) and polydispersity (even 10–20%) of shaped NPs have often hampered its effeciency.^90^ Though requiring multiple-steps for synthesis of shaped gold nanostructures, researchers have been able to drastically improve both reproducibility and scalability. Many different gold nanostructures are now available commercially, confirming the scalability of reproducible production. The ease of surface modification by functionalization with tags,^14,26,71,91^ silica,^15^ polymers,^92,93^ DNA^67,94^ or liposomes^95^ has been a favourable factor for its use. Additionally, a coating of graphene oxide or silica onto gold nanostructures,^96,97^ as well as incorporation of copper selenide (Cu–Se) into gold nanostructures^98,99^ have been employed to boost the absorption cross-section and tune the LSPR into the NIR. Silica and polymer coatings have been used in protecting SERS-tagged NPs providing a stealth layer for frustrating protein corona formation with blood components and inhibiting SERS tag leaching out of the contrast agent into the biological surroundings.^26,84^

Broadband NIR absorbing or resonating gold nanostructures can allow the choice of multiple NIR laser excitations without compromising the performance as a contrast agent. This provides flexibility in instrument design and applications. Examples of such include polycuboctahedral single gold NPs which feature both NIR I and NIR II broadband absorbance, making detection with NIR I and therapy with NIR II feasible. Comparatively broad LSPR peaks in the NIR have been reported for nanobranch assemblies and nanostar morphologies as compared to the distinct sharp and intense longitudinal LSPR peak for nanorods.^100^ Nano-raspberry shaped branched nanostructures (around 36 nm) have also been developed by Sangnier and co-workers^101^ featuring a LSPR peak centred around 600 nm and the absorbance tail extending into the NIR.

Noteworthy are gold nano-assemblies due to their flexibility to modify their morphology and thus their plasmonic properties. Nanobranch assembly morphologies with multiple coupled nanoparticles exhibit significant broadband absorbance. Such assemblies, as opposed to linear nanochain assemblies and single nanorods, provide a NIR I continuum broadband absorbance.^65^ Dey *et al.*^14,102,103^ have reported, in a series of publications, methodologies and factors for controlling the preparation of gold nano-assemblies yielding 1D, 2D as well as 3D morphologies allowing morphology-dependent red-shifted LSPR into the NIR and importantly maximization of NP–NP junction or hot-spot density. In terms of specific nano-assembly morphologies, core-satellite nano-assemblies demonstrate stricter control over their number of NP–NP junctions *i.e.*, hot-spot density and thus plasmon coupling. Such core-satellite morphologies have been reported using branched polymeric linkers with spherical core,^103^ with DNA linkers utilizing gold nanorods as the core,^72,104^ as well as with more complicated structures of caged gold nanorods as core.^105^ Recently, Dey *et al.*^91^ have reported a distinct morphology of core multi-tentacle assemblies that provides the unique features of both core-satellites and branched nanochains. Such nano-assemblies feature an intense broadband absorbance extending throughout NIR I and exhibit dramatically improved NIR absorbance than that of core-satellite nano-assemblies. Although broad LSPR nanostructures may propose a one-size fits all approach due to their broad LSPR allowing freedom of choice in laser excitation, it also suggests variability in the nanostructure which in turn indicates that their reproducibility and scalability might be a concern. Therefore, it is clear that there is huge promise from such nanostructures, but unless issues like scalability, reproducibility and performance standardization are resolved, clinical acceptance is distant.

## 6. Conclusion and future perspective

Coupled with instrumental progress, the development of novel gold nanostructure-based contrast agents has significantly improved the scope and capability of optical imaging and spectroscopic detection. OCT, PAI, MSOT, SESORS have all demonstrated prospects for optical detection of diseased sites with gold nanostructure contrast agents. Tissue absorbance, scattering and auto-fluorescence have tremendous impact on both the light delivered to the nanostructures and the signal travelling back to the detector. To this end, NIR I (650–900 nm) finds extensive use due to overcoming the above limitation, as well as availability of relevant high throughput optics and detectors. Signal retrieval with high signal to noise is achieved with well-engineered optics and use of data analysis, statistical methods and/or using machine learning algorithms to classify data and amplify subtle signal differences. Notably, a major translational barrier for optical imaging to clinical human studies is the limitation of imaging depth that mice of 2–5 cm thickness offers for pre-clincial studies. The possibility of using high-end endoscopic needles with built-in optical detection systems, though more-invasive, might provide an opportunity for in-operation cancer site and tumour margin detection. An interesting multiplexed and synergistic way forward could be the utilization of gold nanostructures for MSOT large-area scanning, followed by SESORS localized disease or tumour-site identification, and subsequently use of local and carefully monitored photothermal therapy to kill the diseased cells, thereby providing an all optical platform with gold nanostructures. On the other hand, design considerations of gold nanostructures indicates the dictating role of LSPR absorbance in improving the detection performance. Especially of interest are the potential of a broader NIR LSPR gold nanostructures due to its “one-size fits all” potential *i.e.*, one nanostructure design may be useful for multiple optical detection modalities. This “one-size fits all” application-specific design approach would need to be debated in order to streamline research focus and accelerate the translational phase. Furthermore, consistency and full disclosure of research methodologies (including but not limited to, laser power, injected gold concentration *i.e.* gold mass, NPs/mL) and other critical parameters are of paramount importance for the clinical prospect of gold nanostructure contrast agents. There is a need for standardized protocols for comparison of detection performance (like SERS enhancement factors *etc.*), as otherwise we are comparing apples to oranges and may be stuck with numbers that are not comparable from one research group to the other, thus leading nowhere. It is therefore of utmost importance that material design and clinical needs go hand-in-hand and dedicated multi-disciplinary teams of material scientists, nano scientists, physicists, engineers, microbiologists, pharmaceutical researchers, histopathologists and clinicians come together as one team to tackle this challenge.

## Conflicts of interest

There are no conflicts to declare.
